# Comment on Numsang et al. (2025) ‘Effects of a culture-specific behavior modification program on glycated hemoglobin and blood pressure among adults with diabetes and hypertension: A randomized controlled trial’

**DOI:** 10.1016/j.ijnss.2025.10.012

**Published:** 2025-10-23

**Authors:** Aaron Aytona Funa, Renz Alvin Emberga Gabay

**Affiliations:** Sorsogon State University, Sorsogon, Philippines

Dear Editor,

The paper “Effects of a culture-specific behavior modification program on glycated hemoglobin and blood pressure among adults with diabetes and hypertension: A randomized controlled trial” [1] shows that an Information-Motivation-Behavioral Skills (IMB)-based, culturally tailored program combining dietary education, context-fit physical activity, and medication-adherence support via interactive classes and a mobile web app achieved significant 12-week reductions in HbA1c and blood pressure versus usual care. In practice, the cultural tailoring comprised Thai/Isan-specific diet guidance (the 6S-6O-1S limits on sugar [e.g., “≤6 tsp sugar, ≤6 tsp oil, ≤1 tsp salt/day], oil/fat, and salt, alongside carbohydrate counting, glycemic index use, and label reading) and Soeng Isan dance to Mor Lam music for activity, while skills training covered correct medication use, individualized goal setting, and device-tracked self-monitoring reinforced by the app, nurses, and peers.

A closely aligned adaptation is warranted in the Philippines, where the burdens of diabetes and hypertension remain substantial. In 2024, an estimated 7.5 % of Filipino adults, about 4.7 million people, were living with diabetes, with further growth projected by 2050 [2]. Hypertension control remains low relative to need, with gaps across diagnosis, treatment, and control among adults aged 30–79 years [3]. Foodways center on *ulam-kanin* (rice-and-dish) meals, with white rice being the most commonly consumed item; coconut oil and sweetened 3-in-1 coffee mixes are also prevalent, patterns that complicate glycemic and blood-pressure control [4].

Beyond diet, Filipino help-seeking patterns in hypertension show delayed contact and plural care. In a longitudinal cohort of low-income households, 26.6 % of hypertensive adults were undiagnosed; after one year, only 25.4 % of those undiagnosed obtained a formal diagnosis. Belief in the effectiveness of traditional medicine and employment were associated with higher odds of remaining undiagnosed. In comparison, monthly blood-pressure measurement and belief in Western medicine were associated with lower odds [5]. These findings support IMB scripts that normalize routine blood pressure (BP) checks, respectfully surface and address beliefs about traditional remedies, and use low-cost community screening to accelerate diagnosis and treatment initiation.

Accordingly, a pragmatic Philippine adaptation can leverage existing primary care workflows without requiring new infrastructure. First, localize the IMB content into Filipino and major regional languages, framing carbohydrate counting around rice portioning (for example, half-plate non-starchy vegetables, quarter-plate rice), swaps to brown or mixed rice, and root-crop alternatives, and translating salt reduction into everyday choices (less patis/bagoong [fish sauce or fermented shrimp paste]; label reading for instant foods). Second, embed brief nurse or Barangay Health Worker counseling during noncommunicable disease (NCD) clinic days, followed by SMS or app nudges for home blood-pressure checks and medication routines, mirroring the trial’s digital reinforcement while fitting barangay realities. Third, set simple targets (glycated hemoglobin [HbA1c] < 7 %; BP < 130/80 mmHg, 1 mmg = 0.133 kPa) with quarterly HbA1c measurements, where available, and point-of-care BP measurements at each visit, using individualized feedback to close adherence gaps. Finally, evaluate the effectiveness through cluster-randomized pilots across barangay health stations and assess whether clinically meaningful HbA1c and BP improvements are sustained at 6–12 months on a regional scale.

A culture-attuned, skills-forward model, such as that of Numsang et al. [1], aligns with Philippine epidemiology, foodways, and observed help-seeking realities. By localizing the package to the *ulam-kanin* (rice-dish-meal pattern) context and directly addressing delayed and plural care-seeking through normalized routine blood-pressure checks, community screening, and respectful engagement with traditional remedy beliefs, nurse-led counseling with light-touch digital supports can connect patients earlier to guideline-based care, improve HbA1c and blood-pressure control, and ultimately reduce cardiovascular risk in Filipino communities.

## CRediT authorship contribution statement

**Aaron Aytona Funa:** Conceptualization; Writing – original draft; Writing – review and editing. **Renz Alvin Emberga Gabay:** Conceptualization; Writing – review and editing.

## Declaration of competing interest

The authors declare there is no conflict of interest.
